# Reduction in thermal conductivity of Bi thin films with high-density ordered nanoscopic pores

**DOI:** 10.1186/1556-276X-8-371

**Published:** 2013-08-30

**Authors:** Gil-Sung Kim, Mi-Ri Lee, Seung-Yong Lee, Jung-Hwan Hyung, No-Won Park, Eun Sun Lee, Sang-Kwon Lee

**Affiliations:** 1 Basic Research Laboratory (BRL), Department of Semiconductor Science and Technology, Chonbuk National University, Jeonju 561-756, Republic of Korea; 2 Department of Physics, National University of Singapore, Singapore 117542, Republic of Singapore; 3 Department of Physics, Chung-Ang University, Seoul 156-756, Republic of Korea; 4 Department of Electrical and Computer Engineering, University of Virginia, Charlottesville, VA 22904, USA

**Keywords:** Bismuth (Bi), Nanoporous structures, 2D thin films, 3*ω* technique, Thermal conductivity

## Abstract

We prepared two-dimensional Bi thin films with high-density ordered nanoscopic pores by e-beam evaporation of Bi metal. For this structure, we used polystyrene beads ranging from 200 to 750 nm in diameter as an etch mask. The typical hole and neck sizes of the Bi thin films with approximately 50 nm in thickness on SiO_2_/Si substrates were in the range of 135 to 490 nm and 65 to 260 nm, respectively. By measuring the thermal characteristics through a 3*ω* technique, we found that the thermal conductivities of nanoporous Bi thin films are greatly suppressed compared with those of corresponding bulk materials. With a decrease in pore size to approximately 135 nm, the thermal conductivity decreased significantly to approximately 0.46 W/m·K at 300 K.

## Background

Thermoelectric (TE) devices can be used for solid-state cooling and power generation from waste heat and environment-friendly refrigeration [1-3]. For the evaluation of their thermoelectric performances, the efficiencies of TE devices can generally be quantified using a dimensionless figure of merit (ZT) or the power factor. ZT is defined as *S*^2^*σT*/*κ*, and the power factor is *S*^2^*σ*, where *S* is the Seebeck coefficient, *σ* is the electrical conductivity, *κ* is the thermal conductivity, and *T* is the absolute temperature. High-performance thermoelectric materials with high ZT values should have a large Seebeck coefficient, high electrical conductivity, and low thermal conductivity. Over the past few decades, bismuth (Bi) and its alloys have been regarded as the most interesting TE material applications at room temperature [4-6] because Bi is semi-metallic with unique electronic properties such as an extremely small carrier effective mass, low carrier density, high carrier mobility, long carrier mean free path, and a highly anisotropic Fermi surface [7]. However, high-performance TE devices with high ZT values have not yet been realized experimentally by employing Bi materials. Recently, for the application in high-performance TE devices, various one-dimensional (1D) nanostructured TE materials, such as nanowires and nanotubes, have been studied widely with the aim of reducing the phonon mean free path [8-12]. Despite the low thermal conductivity of 1D nanostructured TE materials compared with their bulk counterparts, 1D nanostructured materials are not considered suitable for TE devices because their thermal properties depend greatly on the dimensionality and morphology [8-10]. More recently, to overcome these problems inherent of 1D nanostructured TE device systems, several researchers have alternatively studied two-dimensional (2D) thin films [13,14]. In 2010, Tang and co-workers reported that the thermal conductivity of holey Si thin films is consistently reduced by around two orders of magnitude upon the reduction of the pitch of the hexagonal holey pattern down to 55 nm with approximately 35% porosity [13]. Similarly, Yu and co-workers revealed that a Si nanomesh structure exhibits a substantially lower thermal conductivity than an equivalently prepared array of Si nanowires [14]. Accordingly, we believe that 2D nanoporous materials should be promising scalable TE nanostructured materials.

In this report, we present the fabrication of nanoporous 2D Bi thin films, in which high-density ordered nanoscopic pores are prepared by the nanosphere lithography (NSL) technique that we developed previously [15]. The preparation of large-scale nanoporous 2D Bi thin films is based on e-beam evaporation of Bi metal masked by a monolayer of polystyrene (PS) beads (200 to 750 nm in diameter), followed by a reactive ion-etching (RIE) treatment. We successfully demonstrate the thermal conductivity of nanoporous 2D Bi thin films via the four-point-probe 3*ω* method at room temperature [16,17]. The extracted thermal conductivities of the nanoporous Bi thin films are greatly suppressed, relative to those of bulk materials because of the strongly enhanced boundary scattering via charge carriers and bipolar diffusion at the pore surfaces [18].

## Methods

### Fabrication of high-density nanoporous Bi thin films

**Figure 1:**
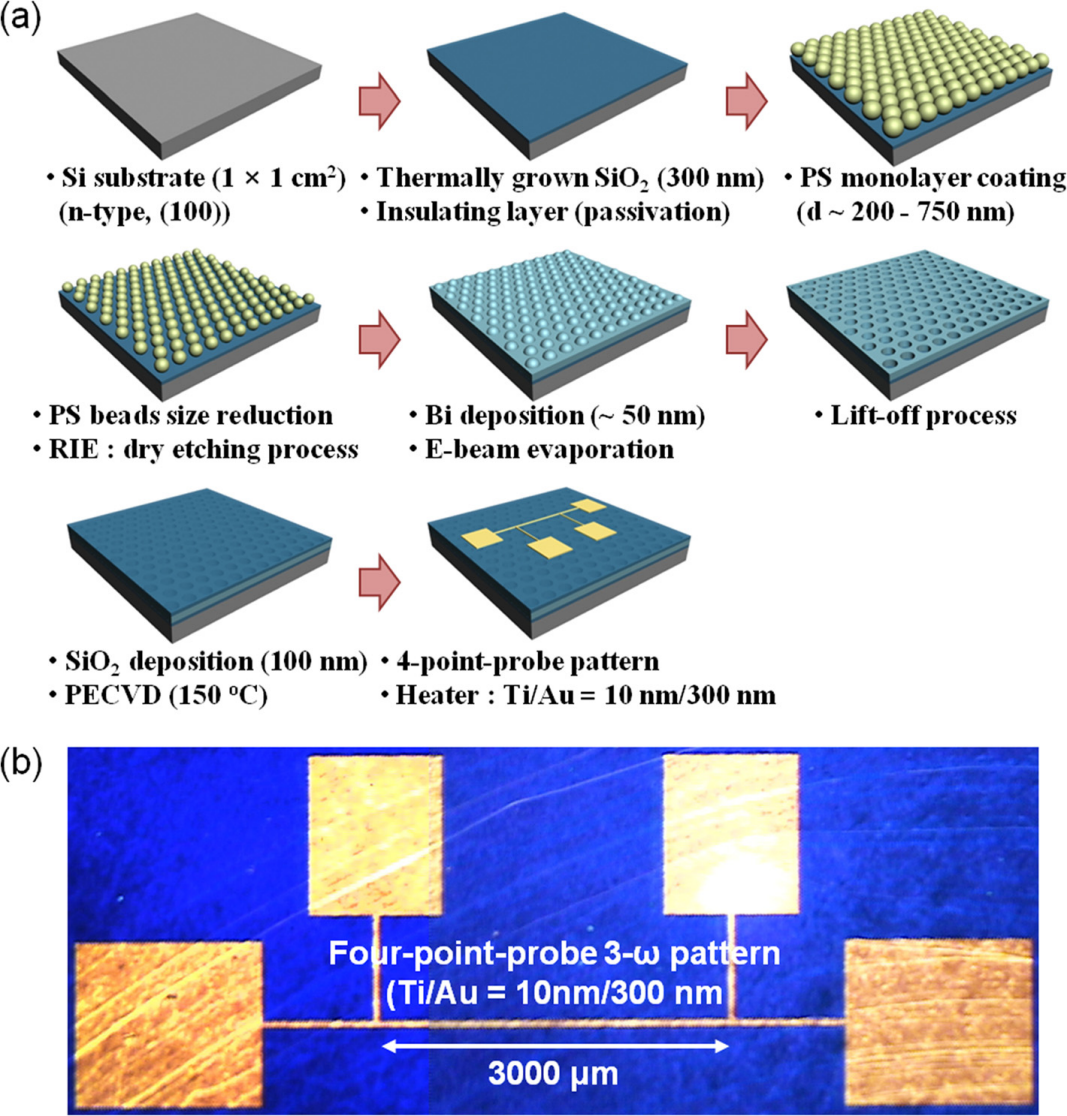
**Diagram of nanoporous Bi samples and image of the narrow metal strips. (a)** Processing diagram of nanoporous Bi samples, consisting of four-point-probe electrodes for measuring the thermal conductivity. **(b)** Optical image of the narrow metal strips (Ti/Au = 10/300 nm) representing the four-point electrodes acting as a heater wire and probe pads.

**Figure 2:**
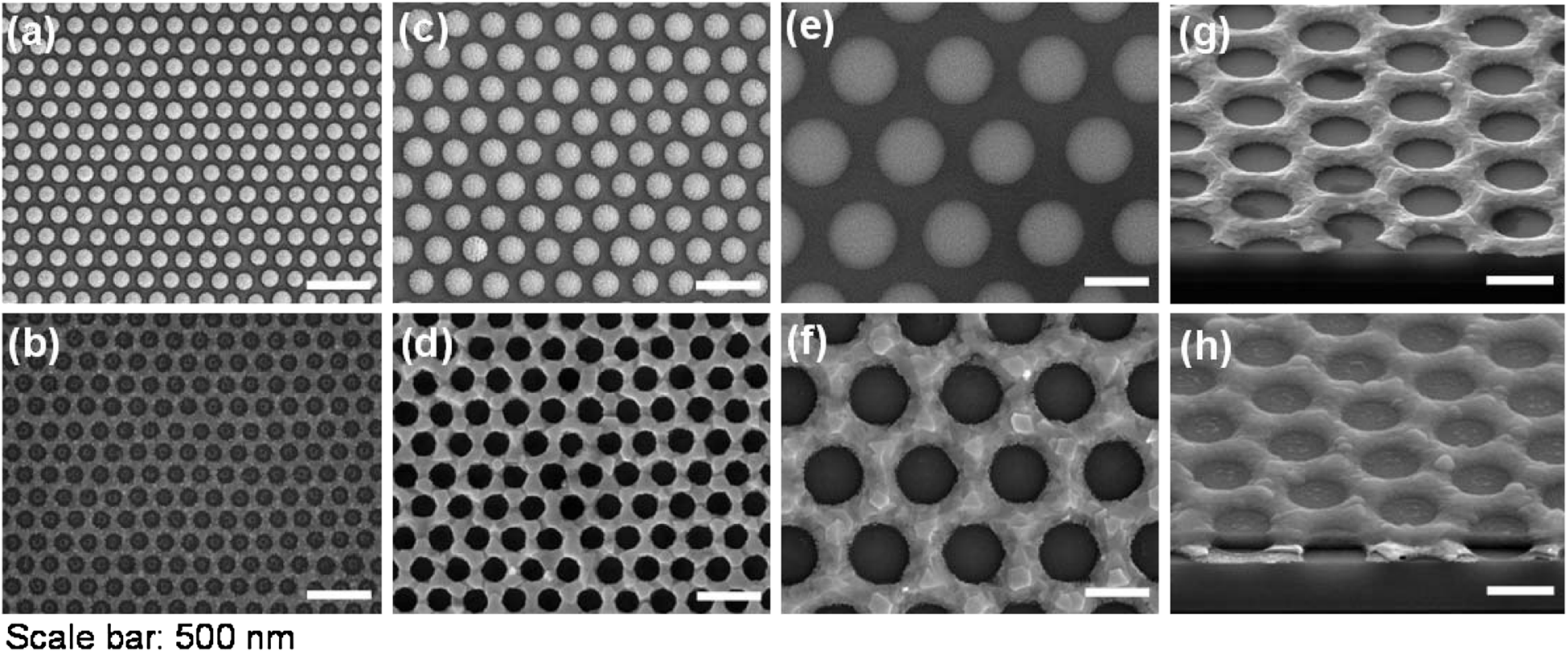
**SEM images of size-reduced PS and porous and nanoporous Bi thin films. (a**, **c**, **e)** SEM images (top view) of size-reduced PS of 200, 290, and 750 nm, respectively. **(b**, **d**, **f)** SEM images (top view) of porous Bi thin films using PS of 200, 290, and 750 nm, respectively. SEM images (tit view) of nanoporous Bi thin films shown in **(f)** before **(g)** and after **(h)** 100-nm-thick amorphous silicon oxide deposition.

Nanoporous 2D Bi thin films were fabricated through NSL and subsequent processes (Figure 1a), as follows. First, PS nanospheres (200 to 750 nm in diameter) were assembled into a hexagonal close-packed monolayer on a water surface through the interface floating method [19]. Subsequently, the PS monolayer was transferred from the water surface to a SiO_2_ (300 nm)/Si substrate. The dried PS monolayers (200, 290, and 750 nm in diameter) were thinned by oxygen RIE (O_2_/Ar = 35/10 sccm, rf power of 100 W, and bias power of 50 W) for 10 s, 200-nm PS; 26 s, 290-nm PS; and 70 s, 750-nm PS, respectively, to control the diameter and spacing of the nanoporous structures during the preparation as shown in Figure 2a,c,e, respectively. Subsequently, a 50-nm-thick Bi thin film was deposited with an e-beam evaporator onto the size-reduced PS nanospheres serving as a shadow mask. This was followed by the dissolution of the nanospheres in toluene, which led to the formation of a highly regular nanoporous Bi thin film (Figure 2b,d,f,g). In addition, the neck size of the nanoporous Bi linearly increased with the O_2_ etching time whereas the hole size of that decreased with increasing the neck size. For electrical insulation of the nanoporous Bi film, a 100-nm-thick SiO_2_ layer was deposited on the Bi thin film through plasma-enhanced chemical vapor deposition as shown in Figure 2h. Finally, a narrow metal strip (Ti/Au = 10/300 nm) consisting of four-point-probe electrodes acting as a heater wire and probe pads was patterned onto the specimen through a conventional photolithography process, as shown in Figure 1b.

### 3ω method for thermal conductivity of nanoporous Bi thin films

**Figure 3:**
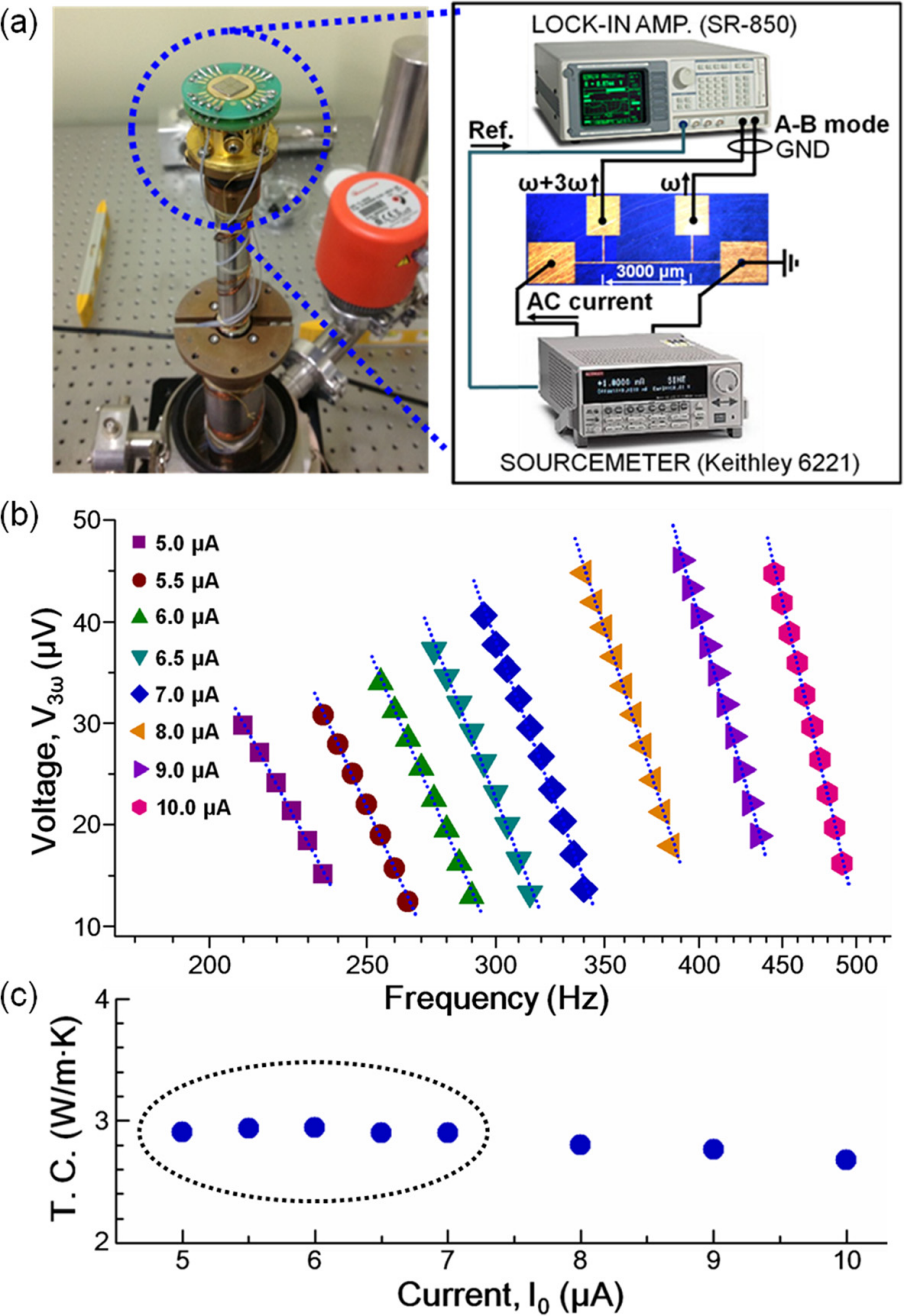
**Thermal conductivities of both nonporous and nanoporous Bi thin films. (a)** Experimental setup and circuit (left side) and corresponding circuit (right side), equipped with thermal management and electrical measurement systems for thermal conductivity measurements via the 3*ω* method at room temperature. **(b)** Linear regions of the third-harmonic voltage versus the applied frequency at various applied ac currents ranging from 5 to 10 μA. **(c)** Thermal conductivities of nonporous Bi thin films in terms of applied ac currents.

To measure the thermal conductivities of both nonporous and nanoporous Bi thin films at room temperature, we used the four-point-probe 3*ω* method (based on the application of an alternating current (ac) current with angular modulation frequency, 1*ω*), which was first developed by Cahill in 1990 [17]. Figure 3a shows the experimental setup including the circuit connections with thermal management and the electrical measurement system for thermal conductivity measurements. The sample was first attached on a printed circuit board substrate (marked by the dashed circle on the left side of Figure 3a) with vacuum grease for mounting inside a closed-cycle refrigerator with a shielding box, as shown on the left side of Figure 3a. The source meter was connected to both metallic pads to apply an ac electrical current (*I*_0_), as shown on the right side of Figure 3a. *I*_0_ with an angular modulation frequency of 1*ω* was applied to generate Joule heat and temperature fluctuations at a frequency of 2*ω*. The resistance of the narrow metal strip is proportional to the temperature that leads to a voltage fluctuation *V* = *IR* of 3*ω* across the specimen. A lock-in amplifier (A − B mode) connected to the two electrodes in the middle receives the 3*ω* voltage fluctuation along the narrow metal strip; that gives the information about the thermal conductivity of the films. A few early studies by our group showed that the thermal conductivities of 1D silicon carbide nanowires (SiC NWs) [16] and Bi NWs [20] were measured successfully with our experimental setup and equipment. For the measurement of the thermal conductivity of nonporous and nanoporous Bi thin films, the third-harmonic voltage (*V*_3*ω*_) must be plotted against the natural logarithm of the applied frequencies ln *ω* resulting in a linear relationship. The thermal conductivity is then determined from the slope in the linear region. Figure 3b shows the linear regions of the plot of *V*_3*ω*_ versus ln *ω* at various applied ac currents ranging from 5 to 10 μA. The characteristic parameters of the linear region calculated from the graphs, as well as other required information, are summarized in Table 1. The difference between two *V*_3*ω*_ values (i.e., *V*_3*ω*1_ and *V*_3*ω*2_) is equated to the temperature drop across the Bi film and is used to calculate the cross-plane thermal conductivity, which is defined by the following Equation:

(1) $$k=\frac{V_{0}^{3}\ln\left(\frac{\omega_{2}}{\omega_{1}}\right)}{4\mathrm{\pi l}R_{0}^{2}\left(V_{3\omega_{1}}-V_{3\omega_{2}}\right)}\frac{\mathrm{dR}}{\mathrm{dT}}.$$

**Table 1:** Summary of the characteristic measuring parameters

***I***_**0 **_**(μA)**	***V***_**0 **_**(mV)**	$\frac{\text{ln}\left(\frac{\omega_{2}}{\omega_{1}}\right)}{V_{3\omega 1}-V_{3\omega 2}}$	***κ *(W/m·K)**	***I***_**0 **_**(μA)**	***V***_**0 **_**(mV)**	$\frac{\text{ln}\left(\frac{\omega_{2}}{\omega_{1}}\right)}{V_{3\omega 1}-V_{3\omega 2}}$	***κ *(W/m·K)**
5.0	564.38	1.76 × 10^4^	2.90	7.0	601.34	1.45 × 10^4^	2.90
5.5	560.23	1.82 × 10^4^	2.94	8.0	627.17	1.24 × 10^4^	2.80
6.0	565.74	1.77 × 10^4^	2.94	9.0	618.19	1.27 × 10^4^	2.76
6.5	607.28	1.41 × 10^4^	2.89	10.0	630.10	1.17 × 10^4^	2.67

The parameters used for the calculation of the thermal conductivity of nonporous Bi thin films as a function of the applied electrical ac current. *R*_*0*_, *dR/dT*, and *l* were determined to be 39.38 Ω, 53.64 mΩ/K, and 3 mm, respectively.

Here, *V*_0_ and *R*_0_ are the applied voltage and electrical resistance, respectively, along the heater wire of length *l*. $V_{3\omega_{1}}$ and $V_{3\omega_{2}}$ are the third-harmonic voltages at input current frequencies of *ω*_1_ and *ω*_2_, respectively, and *dR/dT* is the rate of the resistance change of the heater with its temperature, which fluctuates in the range of 280 to 300 K.

## Results and discussion

Figure 3c shows the thermal conductivity (*κ*) of the nonporous Bi thin film, as calculated from Table 1. When *I*_0_ was 5 μA, the thermal conductivity was determined to be approximately 2.90 W/m∙K at room temperature (300 K). This value is four times lower than that of the homologous bulk material (approximately 11 W/m∙K at 280 K), owing to the strongly enhanced boundary scattering via phonons, charge carriers, and bipolar diffusion induced by the nanoscale crystal grains and the thickness reduction [18,21], which in turn results in a greatly reduced thermal conductivity of the Bi thin films. The detailed phonon thermal transport characteristics (*κ*_ph_), charge carriers (*κ*_e_ and *κ*_h_), and bipolar diffusion (*κ*_eh_) will be discussed in the next section. In particular, *κ* of the Bi films shows similar values in the *I*_0_ range of 5 to 7 μA, whereas it decreases gradually to 2.8, 2.76, and 2.68 W/m∙K with increasing *I*_0_ from 8 to 10 μA. These values are in good agreement with the results of two previous studies reported by Völklein et al., in which it was suggested that the thermal conductivity of planar Bi films of 60-nm thickness was approximately 3.6 W/m∙K at 300 K [22,23]. Thus, our experimental setup and the associated analysis via the four-point-probe 3*ω* method were validated by a comparison with data reported in the literature for nonporous Bi films.

**Figure 4:**
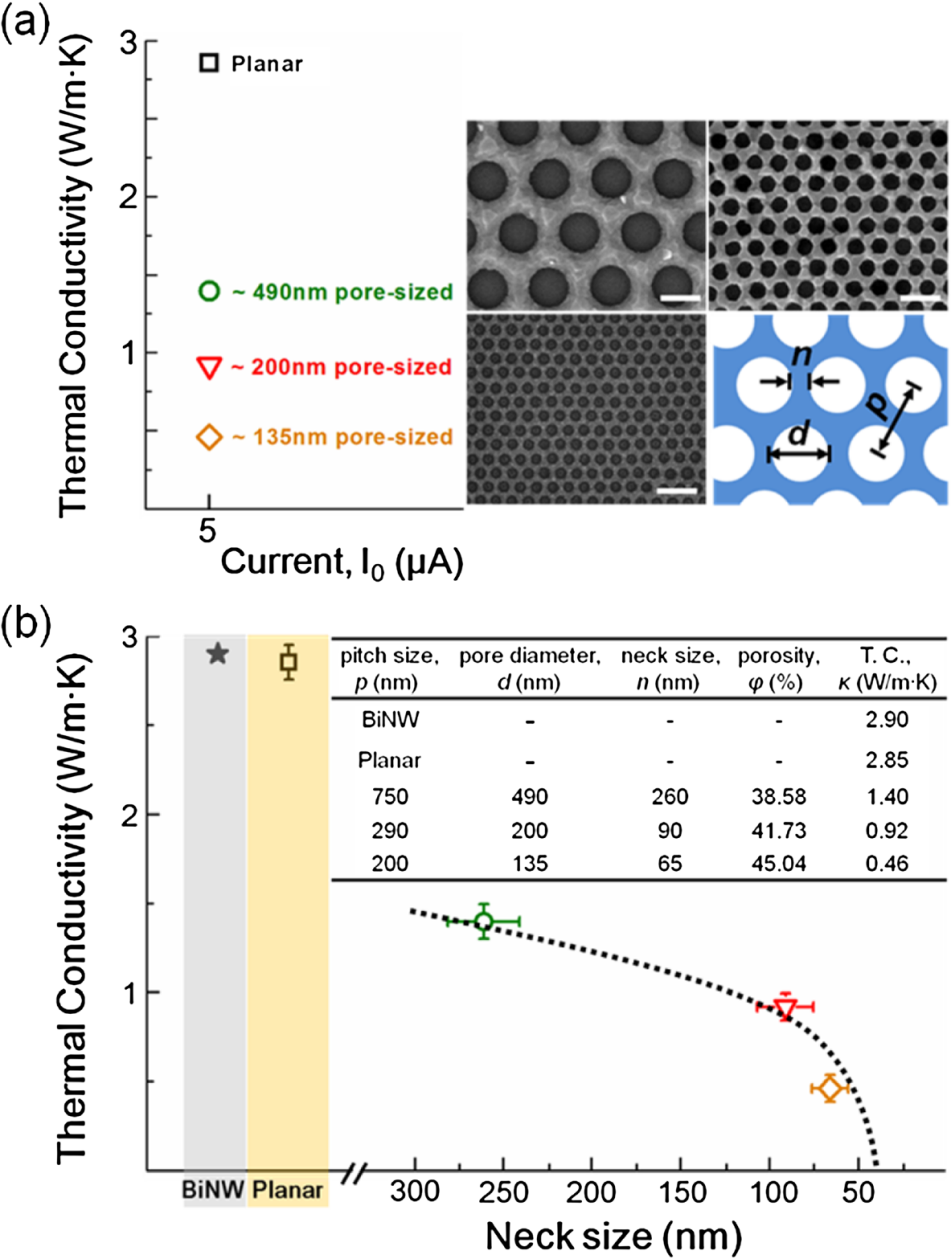
**The thermal conductivities of nonporous and nanoporous Bi thin films. (a)** The thermal conductivities of nanoporous Bi thin films as a function of pore diameters. **(b)** The average thermal conductivities of nonporous and nanoporous Bi thin films plotted against their neck size at room temperature and compared to those of a Bi NW (approximately 123 nm in diameter) at 280 K. Insets show SEM images, and table provides a summary of the geometric parameters of the Bi thin films, *n* is the neck size, *p* is the pitch size, and *d* is pore size, as indicated in the inset. The scale bar is 500 nm.

To investigate the thermal conductivity of the nanoporous Bi thin films, we applied an ac electrical current in the range of 5 to 7 μA to avoid measurement errors. Typical pore diameters of as-prepared 2D Bi films (approximately 50 nm in thickness) on SiO_2_/Si substrates with PS nanospheres with 200, 290, and 750 nm in diameter were determined to be approximately 135, 200, and 490 nm, respectively. While the neck sizes/porosities of the 2D Bi films were approximately 65 nm/45.04%, approximately 90 nm/41.73%, and approximately 260 nm/38.58%, respectively. As shown in Figure 4a,b, the nanoporous Bi thin films exhibit an abrupt reduction in thermal conductivity compared to that of planar films (approximately 2.85 W/m∙K). The thermal conductivity of a Bi sample with 490-nm pore size (approximately 1.40 W/m∙K) is half of that of its nonporous Bi film (flat or planar sample) at 300 K. In addition, the thermal conductivity of a Bi sample of 135-nm pore size was significantly lower with a value of approximately 0.46 W/m∙K. This value is close to that reported by Song et al., who synthesized a nanoporous Bi thin film with a porosity of approximately 26% and a thickness of approximately 62 nm, through metal-organic deposition, and measured the cross-plane thermal conductivity to be approximately 0.81 W/m∙K by using a differential 3*ω* method [24].

For further verification of the correlation between thermal conductivity and neck size, in Figure 4b, the room-temperature thermal conductivities of the three nanoporous Bi films are plotted against their neck size and compared to those of the planar Bi film in Figure 4b and summarized in inset table of Figure 4b. As shown in Figure 4b, the average thermal conductivity shows monotonically decrease by shrinking the neck size up to approximately 65 nm (increasing porosity up to 45.04%). This reduction behavior in thermal conductivity is in good agreement with recent reports of holey Si thin films [13]. Tang et al. reported thermal conductivities of approximately 10.23, approximately 6.96, and approximately 2.03 W/m∙K for holey Si thin films with neck/pitch sizes of 152/350 nm, 59/140 nm, and 23/55 nm, respectively [13]. They also suggested that the thermal conductivity reduction is dominantly influenced by the neck sizes rather than the porosity, by measuring the thermal conductivity of holey Si thin films with different neck sizes (160 to 40 nm) and porosity (13% to 40%). Similarly, Yu et al. demonstrated a very low thermal conductivity of approximately 1.9 W/m∙K at room temperature for a meshed Si structure with neck and pitch sizes of 16 and 34 nm, respectively [14]. Thus, we confirmed that the neck sizes of nanoporous Bi thin films do play the important role in reducing the thermal conductivity.

To elucidate these enormous reductions in thermal conductivity of nanoporous structures, Dechaumphai et al. suggested that phonons be considered as particles in the incoherent regime when the phonon mean free path (MFP) is shorter than the characteristic size of the phononic crystals, and otherwise, phonons be treated as waves in the coherent regime [25]. According to their model, based on the partially coherent effect in phononic crystals, the competition between phonon scattering at pore boundaries in the incoherent regime and the phonon group velocity induced by zone folding effects in the coherent regime leads to an overall monotonic reduction in the total thermal conductivity as the pitch or neck size decreases as shown in Figure 4b. In addition to phonon scattering at pore boundaries, in nanoporous Bi thin films, the contribution of the electronic thermal conductivity (*κ*_E_ *= κ*_e_ *+ κ*_h_ *+ κ*_eh_) should be considered as a key factor due to long charge carrier MFP, which is up to approximately 100 nm for electrons and holes at 300 K [26]. Regarding the contribution of electronic component on thermal conductivity, Gallo et al. reported that approximately 70% of thermal conductivity, at 300 K perpendicular to the trigonal direction, is attributable to *κ*_E_ and the remaining 30% is belonging to *κ*_ph_[7]. Thus, the lattice thermal conductivity is dominant thermal transport at low temperature, whereas the electronic thermal conductivity becomes progressively more important as temperature increase. Similarly, we observed that the thermal conductivity was almost constant up to 200 K and then slightly increased above 200 K in BiNW by enhanced boundary scattering via electrons [20]. As shown in Figure 4b, the length of the charge carrier MFP is longer than the neck size of the nanoporous Bi thin films with approximately 135- and approximately 200-nm pore diameters suggesting that the boundary scattering by charge carriers and bipolar diffusion at the pore surfaces, as the neck size decrease, could play a significant role in the suppression of the thermal conductivity of nanoporous Bi thin films at 300 K. Moreover, the nanoporous Bi thin film exhibits a lower thermal conductivity than 1D Bi NWs. The thermal conductivity of a single-crystalline BiNW (approximately 120 nm in diameter) was measured to be approximately 2.9 W/m∙K at 280 K, confirming that nanoporous Bi thin films exhibit a lower thermal conductivity than 1D Bi NWs [20]. Consequently, the nanoporous architecture should provide promising scalable TE materials with low thermal conductivities, which have advantages over 1D nanostructure, such as nanowires and nanotubes. As a result, we confirm that the enhanced scattering at pore surfaces in such materials can give rise to a significant decrease in thermal conductivity, which, in turn, leads to better thermal properties (ZT) compared with homologous solid thin film and bulk forms. For a better understanding of the thermal transport characteristics of porous Bi films and other porous 2D structures, more detailed studies on the effects of surface morphology, dimensions, and crystalline properties have now been initiated.

## Conclusions

In conclusion, the nanoporous architecture was considered a promising approach to achieve scalable TE materials with low thermal conductivities, which have advantages over 1D nanostructures. To investigate the thermal conductivities of nanoporous 2D Bi thin films, we prepared large-scale specimens using e-beam evaporation of Bi masked using a polystyrene beads monolayer (beads 200 to 750 nm in diameter) and subsequently determined their thermal transport characteristics through the four-point-probe 3*ω* method at room temperature. The thermal conductivity of the Bi thin film of 200-nm pore size was determined to be approximately 0.46 W/m∙K, indicating a significant reduction compared with that in the case of the nonporous sample (approximately 2.85 W/m∙K at 300 K). This might be caused by significant scattering of phonons, charge carriers, and bipolar diffusion as the neck size decreases.

## Competing interests

The authors declare that they have no competing interests.

## Authors’ contributions

GSK carried out the synthesis of 2D Bi thin films with high-density ordered nanoscopic pores by e-beam evaporation. GSK also organized all experiments and prepared the manuscript. MRL and SYL worked the 3*ω* thermal conductivity measurements of 2D nanoporous thin films at room temperature. JHH, NWP, and ESL helped 2D Bi thin film fabrication and thermal conductivity measurements, respectively. SKL finalized data and manuscripts. All authors read and approved the final manuscript.
